# The effect of residency training on arthroscopic knot tying and knot stability: which knot is best tied by Orthopaedic surgery residents?

**DOI:** 10.1186/s40634-018-0138-4

**Published:** 2018-06-15

**Authors:** Kevin J. Cronin, Jacob L. Cox, Timothy M. Hoggard, Scott T. Marberry, Brandon G. Santoni, Charles C. Nofsinger

**Affiliations:** 1 Department of Orthopaedics and Sports Medicine, University of Kentucky, 740 S. Limestone, k403, Lexington, KY 40536 USA; 2 Department of Orthopaedics and Sports Medicine, University of South Florida, 13220 USF Laurel Drive, MDC 106, Tampa, FL 33612 USA; 3 Department of Orthopaedic Surgery, University of Virginia, 400 Ray C. Hunt Drive, Suite 330, Charlottesville, VA 22903 USA; 4 Foundation for Orthopaedic Research and Education, Phillip Spiegel Orthopaedic Research Laboratory, 13020 Telecom Pkwy. N, Tampa, FL 33637 USA

**Keywords:** Arthroscopy, Resident, Education, Resident education, Knot tying, Arthroscopic knots

## Abstract

**Background:**

The aim of this study is to evaluate which of three arthroscopic knots are most reliably taught to and executed by residents at varying levels of training.

**Methods:**

Three arthroscopic knots, the Samsung Medical Center (SMC), the Weston, and the surgeon’s knot, were taught to 16 orthopaedic surgery residents. Each knot was tied in triplicate at two sessions 1 week apart. The knots were then biomechanically tested for strength. Corresponding knots tied by a sports medicine fellow served as the respective controls.

**Results:**

Comparing all knots regardless of year of training, the SMC knot failed at significantly higher loads (237.2 ± 66.6 N) than the surgeon’s knot (203.7 ± 45.3 N, *p* = 0.049) and the Weston knot (193.5 ± 56.1 N, *p* = 0.013). No significant differences in knot strength were found when comparing knots tied by residents at different levels of training and when comparing residents to the sports medicine fellow. There was no difference in conditioning elongation between surgeon’s (*p* = 0.343), Weston (*p* = 0.486), or SMC knots (*p* = 0.200) tied by post-graduate year one and five residents.

**Conclusions:**

We report the first study that evaluates the loop strength of an arthroscopically tied knot performed by orthopaedic surgery residents in various levels of training. In our cohort, the SMC knot required a higher load to failure, when compared to the Surgeon’s and Weston knot, after a simple arthroscopic knot tying curriculum. Based on these findings, he SMC knot should be considered as a part of future orthopaedic surgery resident arthroscopic training programs.

## Background

An increasing number of orthopaedic surgeons are performing arthroscopic surgery. With the rise in popularity of these procedures, residency programs are incorporating arthroscopic knot training into their curricula (Baumgarten and Wright 2007; Fischer et al. 2010; Lo et al. 2010; Loutzenheiser et al. 1998; Milia et al. 2005). Our residency training program, along with many other training programs across the United States, does not have a standardized training protocol for when these knots are to be learned. The residents learn them in no specific order and at no specific time in their training. Few studies have looked into the training methods and evaluation of resident-tied knots (Gilmer et al. 2015; Pedowitz et al. 2015). To date, there is no study that tests the biomechanical loop strength of knots tied by residents in various levels of training after a brief arthroscopic knot tying educational session. Additionally, there are a variety of arthroscopic knots from which to learn (Baumgarten and Wright 2007; Fischer et al. 2010; Lo et al. 2004; Lo et al. 2010; Loutzenheiser et al. 1998; Milia et al. 2005). This creates confusion for orthopaedic surgery residents as well as those attempting to develop arthroscopic knot tying curriculums (Fischer et al. 2010; Gilmer et al. 2015). Establishing which knot is more easily learned by the orthopaedic surgery resident would be beneficial.

Generally, arthroscopic knots can be classified as either sliding or non-sliding. Non-sliding knots are utilized when suture limbs do not slide freely through the tissues being apposed. (Milia et al. 2005). Three commonly used knots at both our training institution and in the literature include the Samsung Medical Center (SMC) knot, the Weston knot, and the surgeon’s knot (Baumgarten and Wright 2010; Fischer et al. 2010; Weston 1991). The SMC and Weston knot are complex locking knots whereas the surgeon’s knot is a non-sliding “static” knot, with a base knot composed of 3 half-hitches followed by a series of 3 reversing half-hitches on alternating posts (Kim and Ha 2000; Lo et al. 2004; Loutzenheiser et al. 1995). The concepts of loop security and knot security are fundamental when evaluating the effectiveness of these arthroscopic knots. Knot security is defined as the ability of a knot to resist slippage with the application of a load, whereas loop security refers to the strength of a first knot after it is locked without three reversing half-hitch throws (Kim et al. 2013; Lo et al. 2010). The purpose of our study was to evaluate the loop strength of the three described knots tied by residents in various levels of training and to identify which knot performs best at various levels of training in regards to loop and knot security. We hypothesize that after a brief educational session, residents at any level of training would be able to tie arthroscopic knots with adequate loop security and knot security. Additionally, we hypothesize that there would be no difference in biomechanical testing between different arthroscopic knots tied by orthopaedic surgery residents.

## Methods

Three of the most common arthroscopic knots in the literature were tested in this study: the SMC knot, the Weston knot, and the surgeon’s knot (Baumgarten and Wright 2010). The SMC and Weston knot are sliding knots while the surgeon’s knot is a static, non-sliding knot. After obtaining Institutional Review Board (IRB) approval, these knots were taught to orthopaedic surgery residents at our institution, the trainees, by a fellowship trained sports medicine orthopaedic surgeon, the instructor. Education consisted of each knot being demonstrated by the instructor with an arthroscopic knot simulator followed by a period of hands on practice with the same simulators. The “SAWBONES” (Pacific Research Laboratories) “FAST” (Fundamentals of Arthroscopic Training) box-trainer was utilized by the instructor to demonstrate the various arthroscopic knots. Each knot was demonstrated by the instructor five successive times while verbally explaining how the knot is tied. The trainees were then given up to thirty minutes to practice all three knots on the box-trainer simulator. The instructor was available during the practice period to answer any questions and assist the residents with knot tying. Formal testing did not begin until the resident felt ready to proceed or thirty minutes of practice had elapsed. Trainees were not formally “approved” to proceed by the instructor and only trainees themselves determined when they were appropriate to begin formal testing. Formal testing consisted of 3 SMC knots, 3 Weston knots and 3 surgeon’s knots during the initial session. Following a one-week washout period the same 9 knots were again tied at a second session. Therefore, each resident tied a total of *n* = 18 knots. A single orthopaedic sports medicine fellow tied the same number of knots to serve as a positive control and allow for a baseline comparison for the study cohort. The knots were tied on an arthroscopic knot-tying simulator using standard arthroscopic portals and knot pusher. Upon completion, the knots were carefully slid off of the arthroscopic testing apparatus without violating the integrity of the knot. The knot tails were then cut to a length of 5 cm and placed into de-identified bags to blind the researchers during biomechanical testing. A braided, nonabsorbent suture material was used for all knots (Ethicon, Somerville, NJ).

**Fig. 1:**
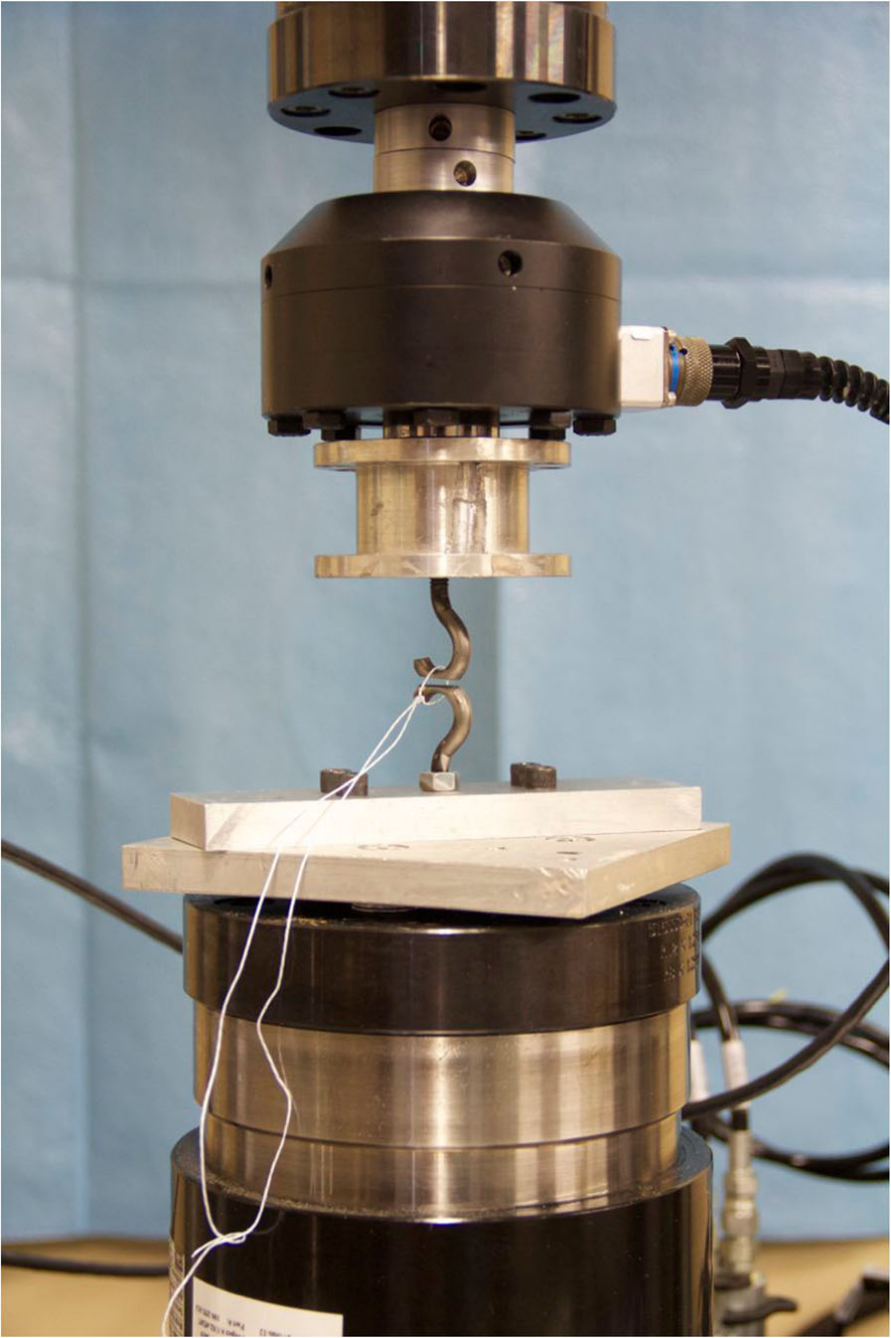
Testing Apparatus. MTS Bionix Servohydraulic Test Frame with suture located between two eyelets

After tying, the intact suture knots were biomechanically evaluated using a servohydraulic axial/torsional test system (MTS Bionix, MTS Inc., Eden Prairie, MN). The knots were secured by looping them between two eyelets rigidly coupled to the test frame and a 10 N preload was applied (Fig. 1) to negate initial slack in the suture loop and serve as a consistent starting reference. Biomechanical evaluation was consistent with models described in the literature and was comprised of two phases: (1) cyclic testing for 25 cycles; and (2) tensile loading to failure (Baumgarten et al. 2008; Loutzenheiser et al. 1995; Loutzenheiser et al. 1998; Mishra et al. 1997). If suture knots failed to withstand the dynamic component of testing, they were recorded as dynamic failures; otherwise, if they survived cyclic loading, they were recorded as a static failure.

### Dynamic cyclic loading

After application of the 10 N pre-load, the knots were then cycled for 25 cycles at 1 Hz from 10 to 30 N. For each knot that survived the dynamic test protocol, the maximal displacement (mm), derived from the output of the MTS actuator’s linear variable differential transformer (LVDT), at the 30 N load level on the last loading cycle was recorded and the difference between the measured displacement on the first cycle and last cycle was regarded as conditioning elongation.

### Load to failure testing

Upon completion of cycling, the knots were then axially loaded to failure at a rate of 1 mm/sec. Force (N) and displacement (mm) were recorded at 100 Hz. Load to clinical failure was defined as the measured load at 3 mm of measured actuator displacement and was based on the accepted definition in literature, which corresponds clinically to the loss of tissue apposition (Baumgarten et al. 2008; Lo et al. 2004; Loutzenheiser et al. 1995; Loutzenheiser et al. 1998; Mishra et al. 1997). Ultimate failure load (N) was defined as the load at which the suture loop failed, either by loop slippage or suture breakage. Maximum force to failure (N) was also recorded at the cross-head displacement point of 3 mm. The mode of failure, knot slippage or knot breakage, was also recorded.

### Statistical analysis

Data are presented as mean +/− standard deviation. Mean conditioning elongation (mm), clinical failure load (N), and ultimate failure load (N) were calculated using the three knots tied per type each week. The pooled variance mean derived from the week 1 and 2 means per knot type was used for statistical analysis. Differences in biomechanical parameters (PGYI vs PGYV) were compared with a non-parametric Mann-Whitney U test using SPSS v22 (IBM, Armonk, NY) at a significance level of α = 0.05 to determine the effect, if any, of post-graduate training. As a final comparison, the clinical and ultimate failure loads for each knot type, derived by creating a single average of all tied knots, were compared using Kruskal-Wallis test and Mann-Whitney U tests for multiple comparisons when necessary.

## Results

The three tested knots were taught to 16 of the 20 orthopaedic surgery residents at our institution by a fellowship trained sports medicine orthopaedic surgeon, the instructor. Four of the twenty residents were excluded due to scheduling conflicts. The cohort consisted of four PGY1, three PGY2, four PGY3, one PGY4, and four PGY5 orthopaedic surgery residents. There were 14 males and 2 females. Knots tied at time zero and at seven days later by all subjects were compared and found to have no difference in failure method or failure rate. Therefore, knots tied at the original training session and after the one-week washout period were grouped together for statistical analysis.

### Dynamic cyclic loading

**Fig. 2:**
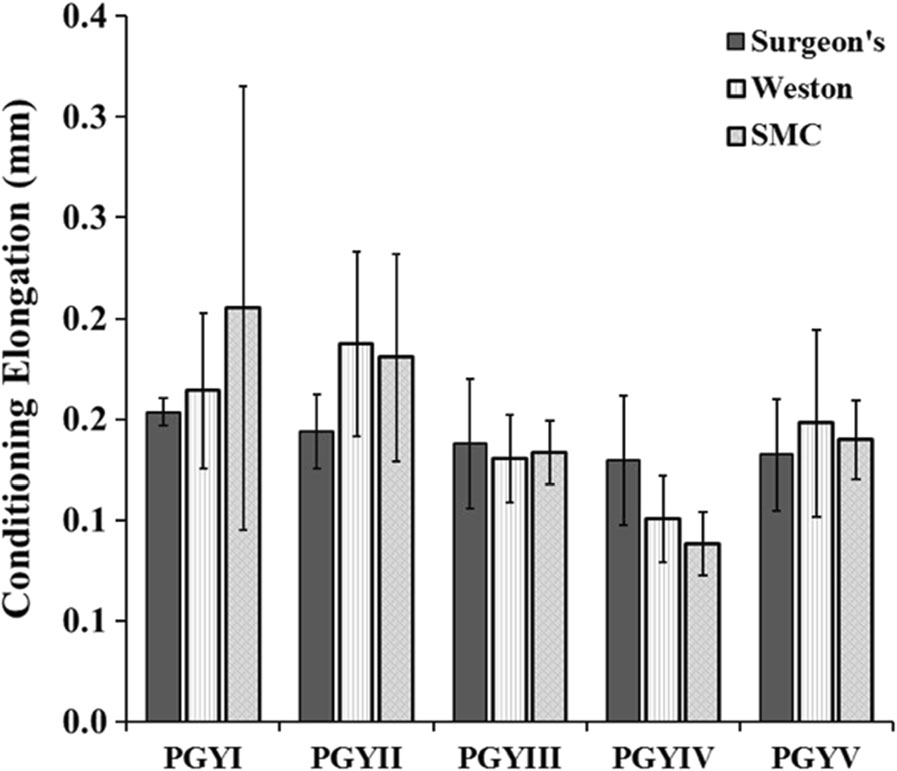
Conditioning Elongation. Conditioning elongation as a function of resident training for the surgeon’s, Weston and SMC knots. Results indicate no significant difference in conditioning elongation for each knot as a function of resident education level (*p* > 0.200)

The Weston knot demonstrated the highest dynamic failure rate (10.4%) during cyclic loading compared with the surgeons (8.3%) or SMC knot (3.2%). There was no apparent effect of resident training on dynamic failure of any knot during cyclic loading. There was no difference in conditioning elongation between surgeon’s (*p* = 0.343), Weston (*p* = 0.486), or SMC knots (*p* = 0.200) tied by PGYI and PGYV residents (Fig. 2). Conditioning elongation of the surgeon’s, Weston, and SMC knots tied by the orthopaedic sports medicine fellow were 0.11 mm, 0.12 mm, and 0.38 mm, respectively.

### Load to clinical failure

Clinical failure loads of the surgeon’s, Weston, and SMC knots as tied by PGYI residents were 103.8 ± 7.9 N, 84.0 ± 10.7 N, and 86.0 ± 28.5 N. For the PGYV residents, clinical failure loads for the same knots were 131.0 ± 19.5 N, 110.1 ± 38.6 N, and 98.4 ± 10.1 N. There was no difference in clinical failure load as a function of resident training for any knot (*p* > 0.114). The clinical failure loads of the surgeon’s, Weston, and SMC knots as tied by the orthopaedic sports medicine fellow were 115.3 N, 112.9 N and 110.2 N, respectively.

When grouping all knots together, the clinical failure loads of the surgeon’s, Weston, and SMC knots were 109.6 ± 28.6 N, 99.6 ± 29.7 N, and 99.4 ± 26.5 N. These differences were not statistically significant (*p* = 0.490).

### Ultimate load to failure

**Fig. 3:**
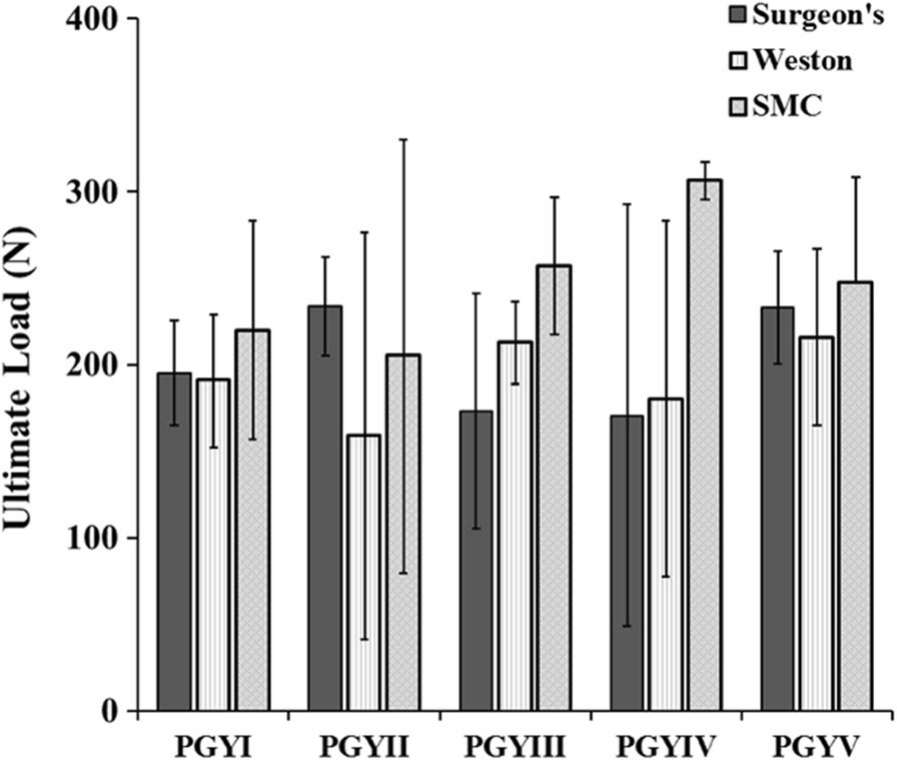
Ultimate Load to Failure. Ultimate failure load as a function of resident training for the surgeon’s, Weston and SMC knots. Results indicate no significant difference in ultimate failure load for each knot as a function of resident education level (*p* > 0.200)

Ultimate failure loads of the surgeon’s, Weston, and SMC knots as tied by PGYI residents were 195.2 ± 30.4 N, 190.7 ± 38.3 N, and 219.9 ± 63.2 N, respectively (Fig. 3). For the PGYV residents, ultimate failure loads for the same knots were 232.9 ± 32.4 N, 215.8 ± 51.0 N, and 247.2 ± 61.4 N, respectively. There was no difference in ultimate failure load as a function of resident training for any knot (*p* > 0.200). The ultimate failure loads of the surgeon’s, Weston, and SMC knots as tied by the orthopaedic sports medicine fellow were 185.8 N, 154.0 N and 215.3 N, respectively.

Comparing all knots regardless of year of training, the SMC knot failed at significantly higher loads (237.2 ± 66.6 N) than the surgeon’s knot (203.7 ± 45.3 N, *p* = 0.049) and the Weston knot (193.5 ± 56.1 N, *p* = 0.013).

Regardless of level of training, 50% of the tied surgeon’s knots failed via suture breakage, while 26% of the Weston and 65% of the SMC knots failed via a suture breakage. When broken down by level of training, 46%, 25%, and 50% of the surgeon’s, Weston, and SMC knots, respectively, tied by PGYI residents failed via suture breakage. In comparison, 71%, 29%, and 71% of the surgeons, Weston, and SMC knots, respectively, tied by PGYV residents failed via suture breakage.

## Discussion

There are a seemingly infinite number of arthroscopic knots discussed in the literature and taught in orthopaedic residencies. Little consensus exists regarding which knots perform the best clinically and which knot a surgeon utilizes may largely be a factor of that surgeon’s specific training. There are multiple factors which contribute to an acceptable arthroscopic knot such as high loop security, high knot security, low profile, and ease of tying. We found the SMC knot to have a higher load to clinical and ultimate failure. This differs from previous results in the literature. Lo et al. utilized a single experienced arthroscopic to tie six different arthroscopic knots and tested them in a similar manner to our study. They found the surgeon’s knot to have the best balance between knot security and loop security with the highest force to failure (Lo et al. 2004).

There is also little consensus as to when and to what degree arthroscopic knot tying should be included in an orthopaedic residency program’s curriculum. Our data suggest that after an adequate educational period is attained, any level of resident is capable of tying acceptable arthroscopic surgical knots. Recent research by Hanypsiak et al. supports this claim (Hanypsiak et al. 2014). When comparing surgeons who perform greater than 200 arthroscopic shoulder cases per year with surgeons who perform less than 200 arthroscopic shoulder cases per year, the authors found no statistically significant difference in ultimate or clinical failure with arthroscopic knot tying. This suggests that the learning curve is small and even inexperienced arthroscopic surgeons, or orthopaedic surgery residents, are able to tie clinically acceptable arthroscopic knots. Therefore, we recommend that as arthroscopic procedures are becoming more ubiquitous, this education should occur early and frequently throughout residency training.

Recently, there has been increasing advocacy for the implementation of an “Orthopaedic Surgery Boot Camp” for first-year residents. These “boot camps” are being trialed at numerous orthopaedic surgery residency programs across North America in various forms. While varying greatly in length, most such sessions include surgical skills training on both cadaveric specimens and simulators. No current consensus is available regarding the appropriate structure, time, or objectives of such “boot camps,” though the literature suggests that they may be of some benefit to first year orthopaedic surgery residents. Some of the most robust data regarding orthopaedic surgery “boot camps” for first year residents is available from the University of Toronto (Sonnadara et al. 2011). While arthroscopic knot tying was not a part of their “boot camp” curriculum, other relevant orthopaedic surgery technical skills, such as wedge cutting using reciprocating and oscillating saws, bone drilling, screw insertion, and plaster splint application, were taught with statistically significant improvements from pre-test measurements. However, the application of these “boot camps” is resource intensive and may ultimately take away from other aspects of clinical training.

While our data suggest that early and frequent arthroscopic knot tying education benefits resident education, there are some limitations with our study. Our sample size is small. While residents were compared by post-graduate training level, we did not account for the specific number of arthroscopic procedures performed by each resident within similar post-graduate training years. We also recognize that our choice of a sports medicine fellow being used as a control as opposed to a sports medicine faculty member may be questioned. However, we know that increased experience does not necessarily equate to increased loop or knot security from the work of Hanypsiak (Hanypsiak et al. 2014). We do not believe that our results would be any different had a sports medicine trained faculty member been chosen to serve as the control.

## Conclusion

We report the first published study that evaluates the loop strength of three different arthroscopically tied knots performed by orthopaedic surgery residents in various levels of training. In our study, knot strength for any of the three knots did not correlate with the level of training of the residents when compared within the different post-graduate levels or against the control knots of the orthopaedic sports medicine fellow. The SMC knot required greater force prior to reaching 3 mm of displacement and failure force when compared to the surgeon’s knot and Weston knot, although this was not significant. We did find that the SMC knot was superior across all levels and all surgeons. Our recommendation is that the SMC knot should be considered as a part of future orthopaedic surgery resident arthroscopic training programs. Studies of a larger scale and with longer follow up are warranted in this situation to further evaluate the efficacy of arthroscopic knot tying training with orthopaedic surgery residents.

## Abbreviations

LVDT: Linear variable differential transformer
PGY: Post-graduate year
SMC: Samsung Medical Center
